# Prevalence of the Pro12Ala missense mutation in the *PPARG2* gene in Kuwaiti patients with primary knee osteoarthritis

**DOI:** 10.4103/0256-4947.75776

**Published:** 2011

**Authors:** Khaled F. Al-Jarallah, Diaa K. Shehab, Mohammad Z. Haider

**Affiliations:** a Department of Medicine, Kuwait University, Faculty of Medicine, Jabriya, Kuwait; b Department of Pediatrics, Kuwait University, Faculty of Medicine, Jabriya, Kuwait

## Abstract

**BACKGROUND AND OBJECTIVES:**

Peroxisome proliferator–activated receptors (PPARs) play an important role in a number of cellular and metabolic functions. This study was carried out to determine the prevalence of a missense mutation (Pro12Ala) in the *PPARG2* gene in Kuwaiti Arab patients with primary knee osteoarthritis (OA) and healthy controls with the aim of identifying a possible association.

**DESIGN AND SETTING:**

A prospective cross-sectional study carried out at three major teaching hospitals (referral centers) in the country over a one-year period.

**PATIENTS AND METHODS:**

The prevalence of *PPARG2* gene Pro12Ala missense mutation was determined in 104 Kuwaiti Arab patients with primary knee OA and 111 ethnically matched healthy controls. The prevalence of this Pro12Ala missense mutation was also determined in clinical subgroups of OA patients divided on the basis of age at onset, function and radiologic grading.

**RESULTS:**

The Pro-Pro genotype of the *PPARG2* gene Pro12Ala missense mutation was detected in 95/104 (91.3%) cases compared to 111/111 (100%) in the control subjects. The heterozygous Pro-Ala genotype was detected in 9/104 (8.7%) of the OA patients, while it was not detected in any of the controls. The Ala-Ala genotype was not detected in any of the OA patients or the controls. No significant differences were detected in the *PPARG2* gene Pro12Ala genotypes in the subgroups of patients classified on the basis of age at onset, functional assessment using Lequesne’s functional index, and radiological grading using Kellgren-Lawrence (K-L) grading.

**CONCLUSIONS:**

This study found no significant association between the *PPARG2* gene Pro12Ala missense mutation and knee OA. However, the presence of the Pro-Pro genotype of the *PPARG2* gene mutation has a protective effect against development of OA.

Peroxisome proliferator-activated receptors (PPARs) are a nuclear hormone receptor super-family of ligand-activated transcription factors. There are a number of subtypes, and the *PPARG2* subtype has been shown to regulate adipocyte differentiation, lipid metabolism and insulin sensitivity. Osteoarthritis (OA) is a prevalent joint disease and is a leading cause of disability. Although the exact etiology and pathogenesis of OA are not fully understood, epidemiological and genetic studies indicate that genetic factors are strong determinants in the onset of OA.^1^,^2^ A genetic contribution to OA has been suggested in several epidemiological studies.^3^ Twin studies, segregation analyses, linkage analyses and candidate gene association studies have generated important information about inheritance patterns and the genome location of potentially causative mutations, although the results across studies have thus far been inconsistent. Linkage and family studies have suggested that there are likely to be both sex-specific and anatomical site-specific genes that influence OA.^3^ Recent evidence suggests that *PPARG2* may have protective effects in OA.^4^ *PPARG2* activation prevents the expression of several inflammatory genes responsible for the pathogenesis of OA, including interleukin-1β, inducible nitric acid synthase, cyclo-oxygenase-2 and microsomal prostaglandin-E2 synthase-1.^4^–^7^ There is also some evidence to suggest that *PPARG2* activation may be chondroprotective by negatively regulating the expression of matrix metalloproteinase-1 and matrix metalloproteinase-13 and by preventing proteoglycan degradation.^4^,^5^,^8^,^9^ It has also been shown that treatment with a *PPARG2* activator, pioglitazone, shows beneficial effects in an experimental model of OA.^10^ Therefore, it has been postulated that *PPARG2* could be a candidate gene for susceptibility to OA. A number of genetic variants in the *PPARG2* gene have been described, the most common being Pro12Ala missense mutation.^11^ In a previous report, Cheng et al^7^ did not find this missense mutation to be associated with OA in a French Canadian population. We carried out this case-control study in Kuwaiti Arab patients with primary knee OA to investigate the prevalence of Pro12Ala missense mutation of the *PPARG2* gene in a different population/ethnic group.

## PATIENTS AND METHODS

This study included patients with primary knee OA and age- and sex-matched healthy control subjects seen in three major hospitals in Kuwait. The inclusion criteria used were primary knee OA and clinical and radiological evidence of knee OA, with no underlying inflammatory disease or malignancy. All patients had a complete clinical evaluation, including demographic characteristics (age, sex), disease-onset age, body mass index (BMI), duration of the disease, functional assessment using Lequesne’s index, and radiological grading using Kellgren-Lawrence grading method (0-4).^12^

During selection of patients for this study, we excluded all patients with a history or family history of collagenopathies. None of the patients had a history of scoliosis or fractures at the time of this report. Twenty percent had osteoporosis and were started on the treatment. This was analyzed further with logistic regression and was not significant. The exclusion criterion with regard to family history of OA was applied based on the information obtained; however, the patient’s family members were not examined. None of the patients had a history of retinal detachment, and none had any surgical intervention for cataracts. Similarly, none of the patients reported hearing problems. In the OA patients, none had skeletal dysplasias or ligamentous hyperlaxity. All of the patients had patellofemoral crepitations and tenderness. None of the patients had restricted pelvic/ knee movements or rotations. Patients with severe knee OA had clinical evidence of bony broadening and lateral tenderness.

The control subjects were adults with similar ethnic backgrounds. All were normal and were seen at the hospital outpatient clinic for minor illnesses. They did not have a history of musculoskeletal system disorders or other diseases with known genetic or hereditary predisposition. The study was approved by the local ethics committee, and informed consent was obtained from all the participants.

### 

#### Genotyping

**Figure 1:**
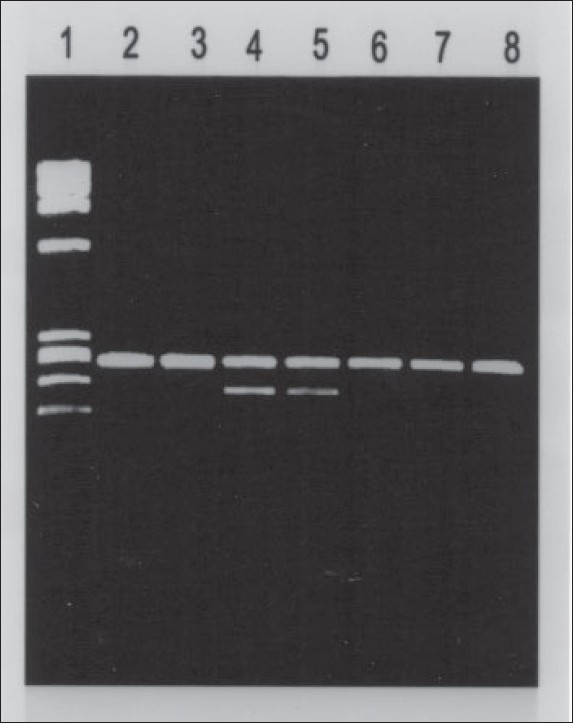
Detection of Pro12Ala missense mutation of the PPARgamma2 gene by polymerase chain reaction–restriction fragment length polymorphism (PCR-RFLP). Lane 1, HaeIII cleaved phiX174 molecular size markers; lanes 2-3 and 6-8, samples with genotype Pro-Pro; lanes 4-5, samples with genotype Pro-Ala. The products of PCR-RFLP analysis were analyzed on 2.5% agarose gel and visualized under UV light following staining with ethidium bromide.

Blood samples (venous) were collected from patients and controls after obtaining verbal consent. The total genomic DNA was isolated by a standard procedure.^13^ The genotypes of the *PPARG2* Pro12Ala missense mutation were determined by polymerase chain reaction-restriction fragment length polymorphism (PCR-RFLP) using primers and conditions described previously.^14^,^15^ PCR reactions were performed with 10 pmol each of sense primer (5’-GCCAATTCAAGCCCAGTC-3’) and an antisense (primer 5’-GATATGTTTGCAGACAGTGT ATCAGTGAAGGAATCGCTTTCCG-3’) that flank the region containing the 12-amino acid site of *PPARG2*.^16^ By using these primers, a PCR product of 270 bp was obtained. The PCR conditions were an initial denaturation step at 95°C for 5 minutes, followed by 30 cycles of denaturation at 95°C for 1 minute, annealing at 56°C and extension at 72°C for 1 minute, with a final extension step of 10 minutes. The PCR products were cleaned using a commercially available spin column and digested with restriction enzyme BstU-I at 60°C for 1 hour. The restriction enzyme BstU-I digests CG-CG only when the C-->G substitution at nucleotide 34 is present. The products of the restriction enzyme digestion were analyzed on a 2.5% agarose gel and visualized after staining with ethidium bromide under UV light. The expected products after digestion with BstU-I were 270 bp for normal (Pro-Pro) homozygous genotype, 227 bp and 43 bp for homozygous (Pro12Ala) genotype and 270, 227 and 43 bp for heterozygous genotype (**Figure 1**).

The clinical data of patients with knee OA was analyzed using SPSS (ver. 17) and STATA (SE 8.2, StataCorp., College Station, TX, USA). The significance of distribution of *PPARG2* gene Pro12Ala missense mutation was determined and compared using the chi-square test. The *P* values were considered significant when they were .05 or less. The genotype frequency followed a normal Hardy-Weinberg distribution.

## RESULTS

This study included 104 patients with primary knee OA. There were 96 females and 8 males, and their mean (SD) age was 56.9 (8.8) years. The study also included 111 healthy controls, 59 females and 52 males, with no significant differences between the two groups in age distribution. The characteristics of the 104 patients, including age, disease-onset age, duration of disease, body mass index (BMI), functional and radiological grades, are shown in **Table 1**. In the OA patients group, the Pro-Pro genotype was detected in 95 (91.3%) of the 104 patients, compared to 111 (100%) of the 111 control subjects (an example of the genotyping is shown in **Figure 1**). The Pro-Ala genotype was detected in 9 (8.7%) of the 104 OA patients studied, while it was not detected in any of the controls. The Ala-Ala genotype was not detected in any of the OA patients or the controls. This data shows that there was no significant difference in the genotype distribution between patients and control subjects for the Pro-Pro and Ala-Ala genotypes (not detected at all). However, the heterozygous Pro-Ala genotype was detected in 8.7% of the OA patients, but not in any of the controls. The clinical profile and characteristics of the OA patients carrying Pro-Ala missense mutation were carefully analyzed and were found to be unremarkable as compared to OA patients carrying the Pro-Pro genotype (data not shown). **Table 2** shows the analysis of association between *PPARG2* gene Pro12Ala genotypes and clinical subgroups by age at onset of the disease (early onset, ≤52 years; and late onset, >52 years). In the subgroups, genotype frequencies showed no significant differences between the OA patients and the controls (**Table 2**). No significant differences were detected in the *PPARG2* gene Pro12Ala genotypes in the subgroups of patients classified on the basis of functional assessment using Lequesne’s functional index and radiological grading using Kellgren-Lawrence (K-L) grading method (**Tables 3 and 4**).

**Table 1:** Clinical characteristics of Kuwaiti Arab patients with primary knee osteoarthritis (n=104).

Clinical features	Values
Average age (year, mean [SD])	56.93 (8.76)
Male/ Female	8/96
Body mass index (kg/m^2^, mean [SD])	31.55 (6.30)
Mean (SD) onset age (years)	51.62 (6.91)
Mean (SD) duration of OA (years)	5.89 (4.97)

**Kellgren-Lawrence grade**	**Number of patients**

1	11
2	40
3	34
4	10
Function (Lequesne’s indices, mean [SD])	10.36 (4.15)

^a^Information not available for 9 patients.

**Table 2:** Prevalence of *PPARG2* gene Pro12Ala missense mutation genotypes in clinical subgroups of Kuwaiti Arab OA patients classified by age at onset of the disease.

	Early onset (<52 years) (n=37)	Late onset (>52 years)	*P*
K-L grade	2.29 (0.87)	2.64 (0.85)	
Functional index	10.22 (5.01)	10.40 (3.63)	
**PPAR-γ2 genotype**			
Pro-Pro	34 (91.9)	42 (91.304)	.622
Pro-Ala	3 (8.2)	4 (8.695)	

Values are mean (standard deviation) or genotype frequency (n,%). The chi-square test was performed to compare *PPARG2* genotype in early-onset OA patients with that in lateonset OA patients. The *P* value was calculated at 2 degrees of freedom. The information on age at onset was not available for some of the OA patients.

**Table 3:** Prevalence of *PPARG2* gene Pro12Ala missense mutation genotypes in clinical subgroups of Kuwaiti Arab OA patients classified by Kellgren-Lawrence grade.

	Severe (Grade 3, 4) n=44	Mild (Grade 1, 2)^a^ n=50	*P*
Mean age at onset (years)	52.95 (6.21)	50.26 (7.52)	
Functional index	12.10 (4.49)	8.94 (3.33)	
***PPARG2* genotype**			
Pro-Pro	40 (90.91)	45 (90.0)	.582
Pro-Ala	4 (9.09)	5 (10.0)	

Values are mean (standard deviation) or genotype frequency (n,%). The chi-square test was performed to compare the *PPARG2* genotype distribution in severe OA patients with that in mild OA patients. The *P* value was calculated at 2 degrees of freedom.

a This information was not available for some of the OA patients.

**Table 4:** Prevalence of *PPARG2* gene Pro12Ala missense mutation genotypes in clinical subgroups of Kuwaiti Arab OA patients classified by Lequesne’s functional index.

	Poor index (n=39)	Good index (n=49)	*P*
Age at onset	53.47 (6.10)	50.53 (6.96)	
K-L grade	2.7 3 (0.76)	2.20 (0.92)	
***PPARG2* genotype**			
Pro-Pro	36 (90.0)	45 (91.836)	.524
Pro-Ala	4 (10.0)	4 (8.2)	

Values are mean (standard deviation) or genotype frequency (n,%). The chi-square test was performed to compare the *PPARG2* genotype distribution in severe OA patients with that in mild OA patients. The *P* value was calculated at 2 degrees of freedom. ^a^This information was not available for some of the OA patients.

## DISCUSSION

Several factors contribute to the risk of developing OA. These include age, sex, genetics, ethnicity, behavioral influences, obesity and occupation.^17^ A genetic contribution to OA has been suggested in several epidemiologic and other studies.^3^,^18^,^19^ The genetics of OA are complex and not completely understood. To assess the validity of reported genetic associations, the best strategy is to reproduce those associations in independent cohorts, preferably focusing on the most clinically relevant phenotypes. Therefore, in this study, we attempted to replicate the results reported earlier by Cheng et al^7^ in a French Canadian population, in a completely different ethnic group—Kuwaiti Arabs.

The data reported in this study showed no significant difference in the distribution of *PPARG2* gene Pro12Ala genotypes between Kuwaiti Arab patients with primary OA and healthy controls. This is in accordance with the previous report among French Canadian OA patients,^7^ which also did not find an association between the *PPARG2* gene Pro12Ala missense mutation and the onset or severity of OA. In our study, the heterozygous Pro-Ala genotype of this missense mutation was detected in 8.7% of the Kuwaiti Arab OA patients, but these patients did not exhibit any unique clinical features in terms of the time of onset or the severity of the disease. In our study, the distribution of *PPARG2* gene Pro12Ala genotypes was not significantly different in the subgroups of OA patients divided on the basis of severity of the disease. This is also in conformity with the results reported among French Canadians,^7^ where no significant association was found between *PPARG2* gene Pro12Ala missense mutation genotypes and the severity of the disease.

In the Kuwaiti Arab patients and controls studied, the Ala-Ala genotype, which represents a homozygous missense mutation in this gene, was not detected in any of the subjects. Although the numbers are relatively small, this indicates that majority of the subjects in Kuwaiti Arab population studied possess the Pro-Pro genotype. The *PPARG2* gene has been implicated in conferring protection against developing symptoms of primary knee OA.^7^ The absence of Pro-Ala heterozygous genotype and the homozygous Ala-Ala genotype of the *PPARG2* gene Pro12Ala missense mutation in Kuwaiti Arab controls supports this previous observation that *PPARG2* gene is indeed protective against the onset of OA. As mentioned earlier, this protective role of the PPAR-receptor G2 gene is possibly due to its influence in the expression of several inflammatory genes responsible for pathogenesis of OA.^7^ Our data in Kuwaiti Arabs certainly supports this protective role of the *PPARG2* gene against the onset of OA. One puzzling question, however, is the presence of a heterozygous genotype (Pro-Ala) in 8.7% of Kuwaiti Arab OA patients without any unique clinical manifestations in terms of the time of onset and/or severity of the disease. The homozygous Ala-Ala genotype was not detected at all in any of the controls. It could be that these 8.7% (9/104) OA patients carrying the heterozygous (Pro12Ala) genotype possess a reduced level of *PPARG2* gene product which is sufficient to confer protection against OA in these patients. Taken together, all these data indicate that *PPARG2* gene has a protective effect against the onset of OA.
